# NanoLC-EI-MS: Perspectives in Biochemical Analysis

**DOI:** 10.3390/ijms241411746

**Published:** 2023-07-21

**Authors:** Natalia Gabrielly Pereira dos Santos, Edvaldo Vasconcelos Soares Maciel, Deyber Arley Vargas Medina, Fernando Mauro Lanças

**Affiliations:** Institute of Chemistry of São Carlos, University of São Paulo, São Carlos 13566-590, Brazil

**Keywords:** biochemical analysis, nano-liquid chromatography, mass spectrometry, electron ionization, metabolomics

## Abstract

Although LC-MS with atmospheric pressure ionization (API) sources is the primary technique used in modern bioanalytical studies, electron ionization mass spectrometry (EI-MS) can provide some substantial advantages over it. EI-MS is a matrix effect-free technique that provides reproducible and comparable mass spectra, serving as a compound fingerprint for easy identification through automated comparison with spectral libraries. Leveraging EI-MS in biochemical studies can yield critical analytical benefits for targeted and untargeted analyses. However, to fully utilize EI-MS for heavy and non-volatile molecules, a new technology that enables the coupling of liquid chromatography with EI-MS is needed. Recent advancements in nanoLC have addressed the compatibility issues between LC and EI-MS, and innovative interfacing strategies such as Direct-EI, liquid electron ionization (LEI), and Cold-EI have extended the application of EI-MS beyond the determination of volatile organic molecules. This review provides an overview of the latest developments in nanoLC-EI-MS interfacing technologies, discussing their scope and limitations. Additionally, selected examples of nanoLC-EI-MS applications in the field of biochemical analysis are presented, highlighting the potential prospects and benefits that the establishment of this technique can bring to this field.

## 1. Introduction

Liquid chromatography coupled with mass spectrometry (LC-MS), particularly with electrospray ionization (ESI) sources, has become the primary analytical technique in biochemical analysis [1]. By combining the separation capabilities of liquid chromatography with the structural information and identification capabilities of mass spectrometry, LC-MS offers a powerful tool for researchers. LC-ESI-MS finds extensive application in targeted and untargeted biochemical studies, playing a crucial role in diverse scientific domains, including medical research, biological genetics, proteomics, lipidomics, metabolomics, and exosome analysis [2,3].

LC-ESI-MS is now a powerful tool for the targeted and untargeted monitoring of biomolecules, endogenous metabolites, therapeutic drugs [1,3,4], and toxicological agents such as drugs of abuse [2]. LC-ESI-MS is becoming increasingly important for early disease diagnosis by identifying diverse metabolites associated with various diseases, including neonatal screening [1]. Recent ambient ionization mass spectrometry developments revolutionize treatment monitoring and surgical operations using “live-MS” technologies [5].

Although electrospray ionization (ESI) has successfully coupled LC and MS, it is important to note that this ionization mode is highly influenced by the composition of the mobile phase and the sample composition. The presence or absence of salts or other interferents can introduce matrix effects, resulting in signal suppression or enhancement [6,7,8,9]. Furthermore, since biochemical analyses involve complex samples, LC-ESI-MS analysis is typically vulnerable to matrix effects due to endogenous compounds (i.e., lipids, proteins, and salts) or exogenous contaminants (e.g., drugs and environmental pollutants). Even when using tandem mass spectrometry (MS/MS) or high-resolution techniques to enhance selectivity, interfering compounds present in biological samples can affect critical method parameters such as limits of detection and quantification, linearity, accuracy, and precision.

ESI is a complex process involving multiple stages, including (i) charge transfer in the liquid phase, (ii) formation of the Taylor cone with multiply charged droplets, (iii) solvent evaporation up to the Rayleigh limit, and (iv) ion release to the gas phase following either the charge residue model (CRM) or the ion-evaporation model (IEM). As a result, matrix interferents, mobile phase additives, and other compounds can affect ionization by altering the solvent viscosity and the droplet evaporation process, forming molecular clusters, and competing or intervening in charge transfer mechanisms, among other effects [10]. The mechanism by which matrix interferents affect the ESI process still needs to be better understood and can be somewhat unpredictable. For example, salts can lead to ion suppression, while lipids can lead to ion enhancement. Due to the unpredictability of matrix effects, variations in response can even be observed among different lots of the same sample or using the same method [10]. Several alternatives have been proposed to address matrix effects in API-MS, including using alternative ionization techniques such as atmospheric pressure chemical ionization (APCI), implementing dedicated sample preparation approaches, and adding deuterated standards. However, alternative API techniques are limited to low-polarity organic analytes and are not entirely free of matrix effects. The sample preparation process is laborious and time-consuming, often resulting in the loss of analytes. Additionally, labeling standards can be costly and not always accessible for all types of target compounds [10].

In contrast, electron ionization mass spectrometry (EI-MS) stands out as a technique practically free of matrix effects, which could be a significant advantage for analyzing biological samples [11]. In EI, ionization occurs in the gas phase, in which the analyte molecules are bombarded with high-energy electrons to produce cationic fragments. Approximately one molecule out of every 10,000 is estimated to undergo ionization in the EI process, which involves intramolecular reactions for ionization and fragmentation. During EI, radical cation species are formed, less prone to proton transfer reactions, eliminating charge transfer between species. Consequently, matrix components have minimal impact on the ionization efficiency and the resulting mass spectra [12].

EI-MS offers exceptional identification capabilities and significant potential for targeted and untargeted studies [13,14]. API techniques are known for generating quasi-molecular and adduct ions. Ionization occurs with minimal or negligible fragmentation, necessitating MS/MS to obtain mass spectra capable of providing structural information. Despite the availability of different fragmentation techniques, collision-induced dissociation (CID) remains the primary method used in MS/MS. However, CID spectra exhibit limited reproducibility between instruments, making them unsuitable for the automated identification of analytes through spectra comparison [15,16]. As a result, the confident annotation of organic compounds in LC-MS untargeted studies using ESI-MS/MS remains a significant challenge, even with the development of advanced computational tools like molecular networks [17,18].

In contrast, one of the critical attributes of EI-MS is its ability to generate highly reproducible and unique mass spectra. The EI-MS spectra of a specific compound serve as its fingerprint and offer much structural information, enabling straightforward identification through automated comparison with spectral libraries or structural elucidation based on well-established fragmentation mechanisms [13,14,19]. Moreover, while extensive research has been conducted on the fragmentation mechanisms of EI-MS, the gas-phase fragmentation reactions subjected to ESI-MS/MS conditions still need further investigation and better understanding [20]. Thus, coupling nanoLC with EI-MS represents a promising alternative for obtaining robust and reproducible mass spectra of LC-amenable compounds. Nowadays, confident annotation of volatile organic compounds can be easily performed through automated EI-MS spectra comparison [21]. Modern nanoLC-EI-MS have the potential to bring similar identification capabilities in the performance of LC-MS untargeted studies. Furthermore, by enhancing the intra-laboratory reproducibility of biochemical LC-MS methods, electron ionization shortens the development time, a highly sought-after characteristic in biochemical analysis [19,22].

EI-MS has been primarily limited to volatile and thermally stable compounds easily analyzable through gas chromatography. Alternative methods are required to transfer heavy and non-volatility molecules to the gas phase, to expand the applicability of EI-MS. One such alternative is the utilization of direct insertion probes, which have been available since the mid-1960s and are commonly found in modern GC-MS instruments [23]. These stainless-steel shafts allow for the insertion of a few micrograms of analyte without breaking the EI-MS vacuum. The analyte can be introduced into a sample vial or crucible, evaporating, or subliming into the ion source. Alternatively, it can be applied as a solution or suspension onto a thin wire loop or pin directly exposed to the electron beam [23]. However, the use of direct EI-MS introduction probes remains largely unexplored, and further studies are necessary to investigate their potential for obtaining EI-MS of proteins, biomolecules, bacteria, or viruses.

Another alternative for introducing non-amenable gas chromatography compounds to the EI-MS source is coupling with liquid chromatography. However, EI-MS operates under vacuum conditions, while conventional liquid chromatography functions with high flow rates of mobile phases. This fundamental difference renders the techniques incompatible. This mismatch can be overcome by reducing the flow rates of liquid chromatography to the nanoL/min range [19]. Over the past decades, significant efforts have been dedicated to overcoming the challenges arising from the disparity between the high-pressure and large mobile phase flow of liquid chromatography and the vacuum environment characteristic of EI-MS [14,19,24,25]. With the advent of nanoLC, this coupling is becoming increasingly efficient and competitive, holding tremendous potential for applications in biochemistry analysis [26].

EI-MS also has the potential to fill the gap between gas chromatography (GC) and LC, allowing the coexistence of GC and LC in a single instrument and enabling unpreceded analysis, such as the determination of non-ESI and non-GC amenable compounds in a single run [27]. These characteristics make EI-MS particularly valuable in various fields, such as lipidomics, metabolomics, and exposome analysis. EI-MS can play a crucial role in these areas by enhancing confident annotation capabilities and improving our understanding of biological systems. Therefore, this review aims to provide a concise overview of the current advancements in nanoLC-EI-MS, highlight its potential applications in biochemical analysis, and discuss future perspectives in this rapidly evolving field.

## 2. The Evolution of LC-EI-MS Interfacing

### 2.1. From Direct Liquid Introduction (DLI) to Modern Liquid Electron Ionization (LEI)

Despite the success of ESI sources, developing efficient LC-EI-MS interfaces remains an ongoing research objective [19,28]. EI-MS has long demonstrated excellent compatibility with GC, making coupling GC and EI-MS one of the most important breakthroughs in modern analytical sciences. Nowadays, GC-MS is a powerful technique that offers high detectability, specificity, and molecular identification capabilities in targeted and untargeted studies of volatile compounds. Conversely, the coupling of EI-MS with LC still entails significant challenges due to the inherent incompatibility between the high-vacuum conditions required for EI operation and the high flow rates of the mobile phase in conventional LC [10]. This incompatibility posed a primary limitation in early LC-EI-MS interfaces, including the direct liquid introduction (DLI), mobile belt (MB), monodispersive aerosol generation interface (MAGIC), and subsequent advancements leading to the particle beam (PB) [29]. Although these interfaces were widely explored in the 1970s and 1980s, their practical applicability was limited by low detectability, a narrow range of analyzable compounds, frequent blockage, and the lack of robustness for routine applications [19,29].

The research conducted by the Cappiello research group in the 1990s provided crucial insights into the compatibility between LC and EI-MS, emphasizing the importance of reducing the influx of mobile phase into the ionization source. These findings led to the development of the Capillary-EI (cap-EI) interface, which emerged as a miniaturized version of the PB interface capable of generating a fine aerosol from capillary mobile-phase flow rates (1–5 µL) using a low flow rate of make-up gas (0.1–0.2 mL/min). This interface enhanced overall sensitivity and markedly improved tolerance for non-volatile buffers by eliminating column eluent splitting [30].

**Figure 1:**
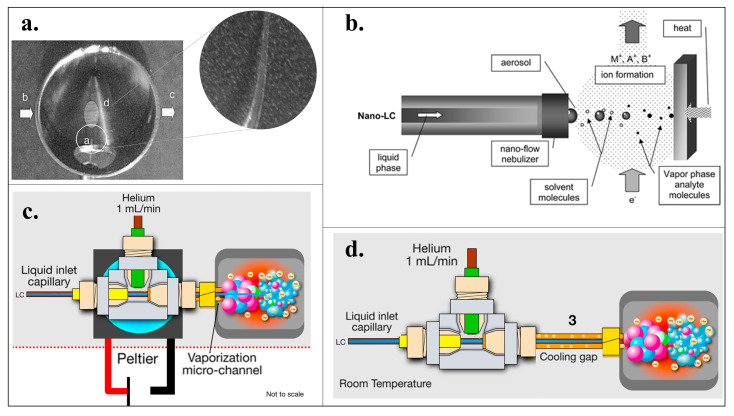
NanoLC-EI-MS interfaces developed in the last two decades. (**a**) Microphotograph of the first version of the Direct-EI interface, showcasing the tapered nebulizer tip (a), the points where the high energy electrons are introduced (b) e (c) and ellipsoidal spot where the formed aerosol jet collides, (d) is the ions formation zone [31]; (**b**) scheme of the Direct-EI interface equipped with nebulization mechanism [36]; (**c**) scheme of the first version of the LEI interface introduced [29]; (**d**) scheme of the more recent LEI interface [29].

In subsequent years, significant advancements were achieved by exploring narrow LC columns with inner diameters ranging from 75 to 100 µm, operating at nL/min flow rates [31]. This breakthrough led to the development of the Direct-EI interface, which directly introduces the nanocolumn effluent into the high-vacuum region of the EI-MS source, eliminating the need for specific solvent removal or make-up gas mechanisms. By employing nanoscale flow rates, the impact of the mobile phase on vacuum conditions and the ionization process is greatly reduced. Consequently, the entire eluent conversion to the gas phase occurs within the ionization source (Figure 1a). Additionally, using nanoLC flow rates (nL) helps preserve the tungsten filaments and can enhance sensitivity by eliminating eluent splitting requirements [32].

In the initial version of the Direct-EI interface, a curved capillary with a cone-shaped tip was employed to enhance the generation of a fine aerosol under high vacuum conditions. This approach successfully achieved efficient analyte ionization, eliminating the occurrence of mobile phase-induced chemical ionization (CI) and mitigating the need for additional hardware components associated with PB and Cap-EI interfaces [31]. In a subsequent advancement of the Direct-EI interface, the complex curved tapered tip was replaced with a segment of the capillary tube (Figure 1b). The internal diameter of the utilized capillary proved to be the critical factor influencing the efficiency of the nebulization/ionization process. Generally, a smaller internal diameter is preferred when the flow rate is lower. Capillaries with a 25 µm internal diameter demonstrated satisfactory performance within the flow rate range of 200–400 nL/min [25].

Despite specific hardware improvements—such as exploring different vaporization surface materials or utilizing dedicated temperature control adapters [33]—having been investigated, the Direct-EI interface is the simplest and most cost-effective LC-EI-MS interfacing strategy presently available. Over the past two decades, the potential of this interface has been demonstrated through a wide range of applications involving both nanoLC and direct sample introduction systems. Direct-EI facilitated the first experimental confirmation of the absence of matrix or mobile phase composition effects in nanoLC-EI-MS. For instance, Table 1 compares the matrix effects observed for ESI-MS and Direct-EI with treating biological samples [25]. In this study, the Capielho research group compared the matrix effects of analytical methods for determining phenacetin and ibuprofen in human blood by LC-ESI-Ms and LC-Direct-EI after liquid-liquid (LLE) and solid phase extraction (SPE). The matrix effects were calculated by performing post-column infusion and post-extraction addition experiments. The absolute matrix effect was calculated as the ratio between the average peak area of the sample spiked after extraction (n = 3) and the average peak area of the neat standard solution (n = 3), multiplied by 100. While in all cases, significantly enhanced/suppression of ionization effects was observed in ESI-MS, the ionization process of direct-EI LC-MS was not affected by matrix effects.

The merits of the nanoLC-EI-MS, featuring a Direct-EI interfacing mechanism, also have been demonstrated through a wide range of applications, including several in the field of biochemical analysis. For instance, Rigano et al. [34] recently employed this strategy to develop a method for elucidating the free fatty acid profile in mussel samples via nanoLC-EI-MS without prior derivatization. The direct connection of a nanocolumn to the EI-MS source through a capillary tubing (27 cm × 25 μm i.d.) via the GC-MS interface allowed the easy fatty acid identification by spectra library comparison.

The Direct-EI interface has proven to be a highly profitable nanoLC-EI-MS interfacing strategy, demonstrating excellent performance in obtaining database-searchable EI MS spectra and facilitating accurate quantitative determinations. However, it should be noted that this setup still encounters challenges in terms of robustness, primarily related to premature solvent evaporation. This issue can result in analyte precipitation and frequent occlusions of the transfer capillary, impacting the reliability and stability of the system. To address these concerns, the Mondello research group, in collaboration with Fasmatech Science and Technology (Athens, Greece), introduced an enhanced version of the Direct-EI interface [19]. A medium-low pressure vaporization/desolvation chamber was implemented in this improved configuration between the column effluent and the EI filament. Nitrogen flow was employed as a make-up gas. At the same time, a heater facilitated the vaporization process, enabling the formed aerosol to be transferred to the ionization source through a pressure gradient [19].

A few years later, the Cappiello research group further improved the interface by incorporating a vaporization micro-channel, enabling the liquid-to-gas transfer outside the ion source at atmospheric pressure (Figure 1c). This innovative approach further led to the development of the liquid electron ionization (LEI) interface, currently the most advanced nanoLC-EI-MS interfacing strategy available [35]. In this setup, a narrow fused silica capillary (25 μm i.d.) serves as the inlet and enters the initial segment of the vaporization micro-channel. The LC eluate is released through this capillary, which swiftly vaporizes upon contact with the hot zone of the micro-channel. Simultaneously, a helium flow aids in accelerating the transport of the vapors into the ion source. A Peltier unit has been introduced at the entrance of the vaporization microchannel to prevent premature solvent evaporation and subsequent analyte precipitation. This addition enables the dissipation of the heat generated by the hot region of the interface.

LEI has demonstrated superior robustness than Direct-EI, showing excellent sensitivity, linearity, and minimal matrix effects. However, the initial version of LEI encountered persistent blockages. To overcome this issue, a cooling gap was introduced between the vaporization and gas-liquid mixing zone, replacing the Peltier unit (Figure 1). This modification effectively reduced eluent preheating, improved temperature control, and significantly minimized clogging of the nebulizer capillary [14]. This latest version of LEI offers reduced hardware and maintenance requirements while enhancing robustness and user-friendliness. This interface represents the optimal approach for nanoLC-EI-MS interfacing, demonstrating a wide range of applicability and delivering excellent results in analyzing pesticides, polycyclic aromatic hydrocarbons, hormones, and phenols [14].

### 2.2. Cold EI with Supersonic Molecular Beams

In parallel and independently, the research group led by Amirav has focused on adapting electron ionization of cold molecules (Cold EI) in supersonic molecular beam (SMB) technology for interfacing LC and EI-MS for the past 20 years. In 2000, the group developed an interface based on high-pressure spray formation, followed by soft thermal vaporization, expansion in a supersonic nozzle, and subsequent ionization [37]. A thermal gradient is created using three heated zones to prevent premature solvent vaporization, and a helium makeup flow assists the supersonic flow. However, the initial version of the SMB LC-EI MS interface encountered issues such as clogging, low robustness, and limited reproducibility [19].

In 2015, Amirav and coworkers introduced a new and improved version of the SMB LC-EI MS interface. This version incorporated a pneumatic spray and a nebulization chamber strategically positioned between the liquid inlet and the vaporization area [38]. The entrance to the vaporization chamber was thermally insulated, allowing for the generation of a low-temperature spray (40 °C), enhancing the stability of the spray. Subsequent vaporization of the effluent took place at a higher temperature (300 °C), and a helium flow toward the SMB nozzle carried the resulting vapors. Vibrational cooling of the molecules then occurred, followed by their conduction to the ion source.

The SMB technology has demonstrated remarkable resilience, effectively handling high eluent flow rates of up to 250 µL min^−1^. It plays a crucial role in promoting molecules’ vibrational cooling, thereby preventing clusters of samples and solvent vapors. As a result, the Cold EI mass spectra exhibit highly pronounced molecular ions, providing significant advantages for molecule identification and greatly enhancing sensitivity. That is particularly valuable since some conventional EI-MS spectra do not display molecular ions, which are crucial for determining the analyte’s molecular mass. Additionally, Cold EI spectra yield enhanced patterns of molecular ion isotope abundance, facilitating the determination of elemental composition using software tools such as the Tal-Aviv Molecule Identifier (TAMI Vr 1.0) software [39].

**Figure 2:**
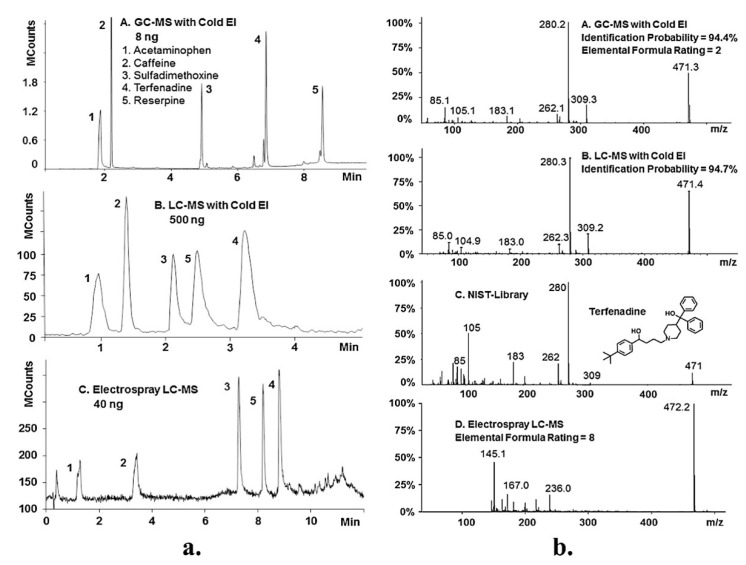
Comparative results using LC-MS with ESI, GC-MS, and LC-MS with Cold-EI in detecting and identifying pharmaceutical compounds [40]. (**a**) Comparative analysis of the obtained chromatograms; (**b**) comparative examination of the obtained mass spectra.

In a recent study, the Amirav research group compared the performance of LC-ESI-MS, LC-MS, and GC with Cold EI (Electron Ionization) to identify pharmaceutical compounds in two model mixtures [40]. Figure 2 displays some key results from this study. Part a of the Figure reveals that while standard GC-MS struggles to analyze terfenadine and reserpine effectively, both GC-MS and LC-MS with Cold EI demonstrate ease in their analysis. Moreover, LC-MS with Cold EI shows competitive detectability compared to LC-MS with ESI—although a fair comparison would require identical injected volumes. Part b of the Figure highlights Cold EI’s ability to generate library-searchable EI-MS spectra for both GC-MS and LC-MS. Notably, the identification probability of terfenadine using Cold EI-MS with EI-LC-MS reaches an impressive 94.7%. In contrast, the ESI-MS analysis of terfenadine mainly relies on a dominant M + 1 ion at *m*/*z* = 472, providing limited structural information due to the predominance of low-mass ions associated with baseline noise.

Amirav’s research group asserts that Cold EI surpasses standard EI as a superior alternative. Unlike CI and other “soft” ion sources, which complement standard EI, Cold EI can fully replace EI and effectively analyze molecules with varying polarity and molecular weight without requiring additional ionization modes. Cold EI offers the advantage of providing both standard EI spectra without any instrumental modifications and enhanced Cold EI spectra for enhanced analysis [41].

The exceptional capability of EI-MS to integrate GC, LC, and even supercritical fluid chromatography into a single instrument is genuinely remarkable. Recently, Amirav’s research group introduced an innovative instrument that combines GC-EI-MS and LC-EI-MS [13]. This cutting-edge instrument enables seamless switching between the two modes (GC and LC) using the same software without hardware modifications. Moreover, it allows for the acquisition of GC-MS and LC-MS data within a single chromatogram, with the flexibility to employ conventional 70 eV. EI-MS or Cold EI. The authors successfully demonstrated the remarkable feat of obtaining highly reproducible and comparable EI spectra through GC-MS and LC-MS. Although this capability is currently limited to high analyte concentrations, it represents a significant advance in developing unified GC/LC instruments. Such instruments can potentially reduce costs in analytical facilities and offer valuable insights for method development.

## 3. Potential of NanoLC-EI-MS in Biochemical Analysis

Despite notable advancements in nanoLC-EI-MS in recent years, there needs to be more literature on comprehensive reports using LC-EI-MS for biochemical analysis. This situation can be attributed to the underdeveloped nature of the technique and its limited accessibility, as only a few research groups worldwide are actively working in this area. These dedicated groups are focused on developing various coupling interfaces and exploring their applications. Therefore, this topic aims to provide compelling examples of LC-EI-MS applications, specifically focusing on biochemical-related analysis. The advantages and disadvantages of LC-EI-MS compared to conventional analytical techniques, particularly LC-ESI-MS, will be highlighted, shedding light on the unique capabilities and challenges in biochemical analysis. Table 2 summarizes selected examples of biochemical analysis applications using LC-EI-MS, encompassing various interfaces.

NanoLC-EI-MS has been tested for the determination of pharmaceutical drugs used to treat psychiatric disorders (such as depression and anxiety) or as painkillers (including analgesics or anesthetics). The use of EI in NanoLC-EI-MS offers the advantage of minimal signal interference from undesired matrix components in the MS spectra. This potential advantage of using EI instead of ESI could lead to shorter analysis times by reducing the time required for sample preparation in biological analyses [48]. Two interesting examples of nanoLC-EI-MS publications of faster analytical methods that avoided intricated sample preparation were: the determination of benzodiazepines in alcoholic beverages [49] and the employment of a nanoLC-EI-MS with a Direct-EI interface to study in-vitro absorption of trans-Cinnamaldehyde in dermal tissues from humans and animals [47]. While these studies may not be directly related to biochemical analysis, they provide valuable insights into analytes and matrices of interest to clinicians in this relatively unexplored field. Another exciting application with potential interest in the biochemical analysis field was published by Cappiello et al. [46]. Their study utilized a nanoLC-EI-MS system to identify potential genotoxic impurities (PGIs) in active ingredients employed for drug formulation. This investigation highlights the time-saving benefits achieved by bypassing extensive sample preparation and derivatization processes that would likely be required when employing LC-ESI-MS or GC-MS techniques.

In addition to the lower time spent in sample preparation due to the negligible matrix effects of the nanoLC-EI-MS systems, the characteristic hard-ionization process is another attractive advantage of this technique for biochemical analysis [19]. The 70-eV energized electron beams accountable for fragmenting target analytes are so intense that such a process generated MS spectra with a well-recognized ionization pattern, essential in untargeted studies where elucidation of unknown compounds or discovery of biomarkers is pursued. By generating reliable and reproducible MS spectra, modern nanoLC-EI-MS systems produce library-matchable data easily compared with available information from different EI-MS spectra sources. Taking advantage of such positive features, Rigano et al. [34] published a method based on nanoLC-EI-MS for profiling mussels’ free-fatty acids (FFAs). It is an important precedent for the further development of studies in lipidomics involving nanoLC-EI-MS. In this case, the prior-derivatization step commonly taken for traditional FFAs analysis by GC-MS was unnecessary, while obtained library-matchable data were further used as an aquatic environmental indicator in biomonitoring studies.

Similarly, Trufelli et al. conducted a study using nanoLC-EI-MS to determine the profile of non-esterified fatty acids (NEFAs) in human plasma [45]. Derivatization steps were not required, and the obtained mass data, which were library-matchable, were used as a health indicator in diabetic patients. Two other examples of the application of nanoLC-EI-MS in biochemical-related studies were reported in papers that described advancements in the EI source, leading to improvements in MS spectra. These studies focused on analytes of interest in biochemical analysis, such as steroids [50] and caffeine [33].

Although current laboratory-based LC-MS systems perform well in most cases, there is a growing tendency to develop faster and more portable LC-MS instruments to carry out biochemical or toxicology exams in the field (e.g., a surgical room). That represents another application where nanoLC-EI-MS can be highly advantageous. The combination of nanoLC and EI-MS appears to be an ideal solution for developing portable instruments. Unlike GC, which requires high temperatures and bulky ovens, nanoLC-EI is a technique that can be more easily implemented in a compact design. That is especially true if it is adaptable to nano-open tubular liquid chromatography (OTLC), which can operate with simpler and more miniature pumps [10,51]. Jocelyn and colleagues featured a fully portable nanoLC-EI-MS system [52] that employed a 15 µm i.d. × 50 cm long fused silica transfer line to connect an Easy-nLC 1000 with a Viking 573 Mass spectrometer. During field testing for the detection of psychoactive substances, the system achieved LODs on the order of 1.5 ng mL^−1^. In our humble opinion, this portable instrument still needs significant instrumental improvements to be considered fully portable. However, this does not diminish the author’s contribution by showing the possible directions toward developing portable nanoLC-EI-MS instruments, which might be extremely useful in assisting other biochemical-related assays.

**Figure 3:**
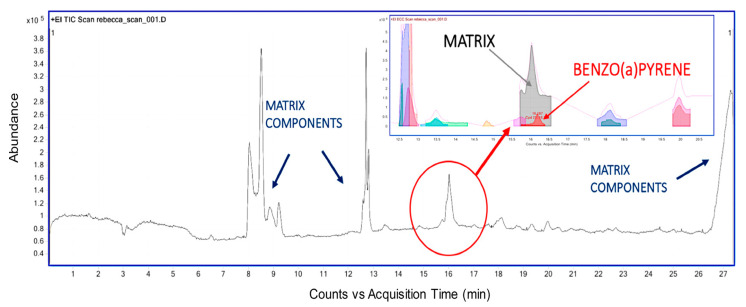
Application of the LEI interface’s distinctive identification capabilities for the analysis of an extract derived from a brain sample of a sudden infant death syndrome (SIDS) victim. The chromatogram reveals the identification of benzo[a]pyrene beneath a prominent matrix peak facilitated using a deconvolution program [35].

LEI is highly regarded for its ability to detect non-targeted compounds, a feature further enhanced through advanced deconvolution software. This software is crucial in reducing background noise and isolating individual mass spectra acquired during sample elution. For example, the Cappiello research group explored the potential of this technique in determining toxic environmental contaminants in a minute brain tissue sample from a sudden infant death syndrome (SIDS) victim [35]. A complex chromatogram displaying multiple peaks originating from brain matrix residues was obtained after extraction and analysis using LEI in full scan mode. However, by employing deconvolution software (Agilent Mass Hunter Unknown Analysis 7.0), a distinct signal emerged beneath a prominent matrix peak, conclusively identified as benzo[a]pyrene (Figure 3). This outcome effectively demonstrates the unique and straightforward identification capabilities of LEI interfacing, particularly when analyzing highly complex biological samples.

An additional area where multiple studies utilizing LC-EI-MS have been reported is the analysis of dietary supplements marketed as health-supporting products. Although these applications may not specifically employ capillary or nanoLC, they serve as valuable examples for researchers interested in exploring the potential of LC-EI-MS in these areas. As public awareness regarding healthy lifestyles and the consumption of natural supplements continues to grow [44], it becomes crucial for regulatory bodies to ensure these products’ standardization and quality control. Analytical chemistry plays a vital role in this context, providing methods for sample extraction, preparation, separation, and detection of the target compounds [53]. However, characterizing active components in natural supplements poses a significant challenge. With the growing consumption of these products by individuals, there is a need for advanced methods to gain a deeper understanding of their associated benefits by providing selective structural information about the active compounds. In this regard, a hard ionization mode like EI-MS can offer valuable insights and perform exceptionally well in such analyses.

Zhang et al. [44] conducted a study focused on quantifying the concentration of five soy-content isoflavones (daidzin, glycitin, genistin, daidzein, and genistein) often used as a dietary supplement with widely known potential sound effects over heart diseases, and some cancers. These active compounds are typically analyzed using conventional LC coupled with ultraviolet-visible light detection (UV-vis). However, the absorption characteristics of isoflavones overlap with other compound classes commonly found in botanical materials, resulting in poor method selectivity. The authors proposed using LC-EI-MS with a particle-beam interface to address these limitations. In their study, neat solutions of single isoflavones were analyzed using LC-PB-EI-MS, employing full scan and selected ion monitoring (SIM) chromatograms and resulting mass spectra to validate retention time assignments, mass spectral characteristics, and chromatographic resolution of each component. Subsequently, in analyzing complex real samples, analyte identification was based on the mass spectral features at individual retention times. At the same time, quantitation was performed by integrating peaks in the extracted ion chromatograms for each isoflavone. The results obtained using the LC-PB-EI-MS method were compared with certified and reference values for NIST SRM 3238 Soy-Containing solid oral dosage form to validate its accuracy and precision. The LC-PB-EI-MS approach consistently provided results in agreement with the NIST-certified or reference values and their uncertainties for all five isoflavones. Moreover, the LC-PB/EIMS approach offers simultaneous structural information and accurate and precise measurements for determining the target isoflavones.

Another interesting report with the same focus and also using LC-EI-MS with a PB interface was published by Burdette et al. [53]. In this specific case, we would like to raise attention to another advantage of LC-EI-MS, of course, without excluding the abovementioned qualities that are also present in this work. Herein the authors analyzed isoflavone presence in dietary supplements based on three distinct natural sources (i.e., Soy, Red Clover, and Kudzu), and in all cases, target analytes exhibited reproducible and accurate results. The central point to underscore here is that they do not perform any complex sample preparation step or hydrolysis before the LC-EI-MS analysis of any sample. Other works dedicated to studying the composition of dietary supplements by using LC-EI-MS include the determination of selenium (Se) in supplements produced from yeast [54]—additionally to the analyzes performed in the supplement, the authors also used LC-EI-MS to analyze urine from patients treated with such a product; the quantification of naturally-occurring ephedrine alkaloids in standard reference materials used to support pharmaceutical developments of drugs mainly to treat fever, cough, and asthma [43]; and the quantification of polyphenols and caffeine from *Camellia sinensis* extracts certified as NIST reference materials used to support surveillance of dietary supplement quality control [42].

## 4. Conclusions

ESI is widely recognized as the primary ionization technique used in LC-MS coupling, offering exceptional sensitivity and performance for analyzing both large biomolecules and small molecules in general. However, LC-ESI-MS still possesses certain limitations. It is susceptible to mobile phase composition and matrix effects and is restricted to polar or ionizable compounds. The fragmentation of small molecules in LC-ESI-MS/MS under varying fragmentation energies remains incompletely understood. In untargeted studies, the limited reproducibility of CID mass spectra on different instruments poses a significant challenge for the confident annotation of organic compounds using ESI-MS/MS.

In contrast, EI-MS has the potential to address these limitations. The vacuum ionization conditions inherent in EI-MS minimize the influence of mobile phase or sample composition on the ionization process, ensuring high reproducibility of obtained spectra across different instruments and facilities. GC-MS spectra can now be interpreted automatically through database searches, benefiting from the collection of reference spectra over many years and the reproducibility of fragmentation mechanisms on different instruments. The coupling of nanoLC with EI-MS presents an opportunity to leverage these advantages in LC-MS analysis.

Significant progress has been made in recent years in bridging the gap between nano-LC and EI-MS. Over the past two decades, advancements in instrumentation have facilitated the development of nanoLC-EI-MS techniques, utilizing interfaces such as Direct-EI or LEI. These innovative approaches have exhibited excellent analytical performance in various applications, including some bioanalytical related, such as the targeted and untargeted determination analysis of drugs, metabolites, and toxicants [14].

Although nanoLC-EI-MS is still undergoing further development, advancements in LC miniaturization, such as the emergence of 2 µm i.d. OT columns capable of operating at pL min^−1^ flow rates, hold the potential to establish nanoLC-EI-MS coupling as a routine technique in the future [55,56].

## Figures and Tables

**Table 1:** Relative matrix effect (ME) evaluation in human plasma using liquid chromatography (LC)–electrospray ionization (ESI)–tandem mass spectrometry (MS) and direct electron ionization (EI) LC-MS [25].

Compound	Summary of the Experimental Conditions		ME ± RSD (%)	
	Matrix Investigated	Extraction Method	ESI-MS	Direct-EI LC-MS
Ibuprofen	Human plasma	LLE	64 ± 22	101 ± 5
	Human plasma	SPE	52 ± 14	97 ± 4
Phenacetin	Human plasma	LLE	135 ± 14	99 ± 2
	Human plasma	SPE	123 ± 13	101 ± 2

**Table 2:** Selected examples of biochemical analysis applications using LC-EI-MS.

Matrix	Analytes	Mobile Phase Flow Rate (µL min^−1^)	Interface	Acquisition Mode	LODs	Ref.
Green tea NIST standard reference	catechins	900	PB	TIC and SIM	5.8–74 ng mL^−1^	[42]
Botanical extracts	ephedrine alkaloids	1000	PB	TIC and SIM	<1.0 ng mL^−1^	[43]
Botanical extracts	Isoflavones	1200	PB	TIC	--	[44]
Human plasma	fatty acids	0.4	Direct-EI	TIC and SIM	1–12 pmol	[45]
Mussel tissues	fatty acids	0.15	Direct-EI		0.19–2.25 µg mg^−1^	[34]
Acetaminophen 500 mg tablets	potential genotoxic impurities	0.4	Direct-EI	SIM	0.13 to 1.5 μg g^−1^	[46]
in Vitro Skin Penetration Samples	trans-Cinnamaldehyde	0.4	Direct-EI	SIM	0.1 ng μL^−1^	[47]
Human Brain	Untargeted	0.1	LEI	TIC	--	[35]

## Data Availability

Data sharing not applicable. No new data were created or analyzed in this study. Data sharing is not applicable to this article.

## Abbreviations

APCI	Atmosphere pressure chemical ionization
APPI	Atmosphere pressure photoionization
Cap-EI	Capillary-EI interface
CRM	charge residue model
CID	Collision-induced dissociation
Direct-EI	Direct-EI interface
DLI	Direct liquid introduction interface
EI	Electron ionization
ESI	Electrospray ionization
FFAs	free-fatty acids
GC	Gas chromatography
HPLC	High-performance liquid chromatography
HRMS	High-resolution mass spectrometry
IEM	Ion-evaporation model
LC	Liquid chromatography
MAGIC	Monodispersive aerosol generation interface
MB	Mobile belt interface
MS	Mass spectrometry
MS/MS	Tandem mass spectrometry
*m*/*z*	Mass-to-charge ratio
NEFAs	non-esterified fatty acids
NIST	National Institute of standards and technology
PGIs	Potential genotoxic impurities
PB	Particle beam interface
SMB	Supersonic molecular beam
UV-vis	Ultraviolet detector
